# Natural Polyphenols—Resveratrol, Quercetin, Magnolol, and β-Catechin—Block Certain Aspects of Heroin Addiction and Modulate Striatal IL-6 and TNF-α

**DOI:** 10.3390/toxics11040379

**Published:** 2023-04-17

**Authors:** Shaimaa ElShebiney, Rania Elgohary, Marwa El-Shamarka, Noha Mowaad, Osama A. Abulseoud

**Affiliations:** 1 Department of Narcotics, Ergogenics, and Poisons, National Research Centre, Dokki, Cairo 12622, Egypt; 2 Department of Psychiatry and Psychology, Mayo Clinic, Phoenix, AZ 85001, USA; 3 Department of Neuroscience, Graduate School of Biomedical Sciences, Mayo Clinic College of Medicine, Phoenix, AZ 85001, USA

**Keywords:** heroin, conditioned place preference (CPP), naloxone precipitated withdrawal, polyphenols, resveratrol, magnolol, β-catechin

## Abstract

We have examined the effects of four different polyphenols in attenuating heroin addiction using a conditioned place preference (CPP) paradigm. Adult male Sprague Dawley rats received heroin (alternating with saline) in escalating doses starting from 10 mg/kg, i.p. up to 80 mg/kg/d for 14 consecutive days. The rats were treated with distilled water (1 mL), quercetin (50 mg/kg/d), β-catechin (100 mg/kg/d), resveratrol (30 mg/kg/d), or magnolol (50 mg/kg/d) through oral gavage for 7 consecutive days, 30 min before heroin administration, starting on day 8. Heroin withdrawal manifestations were assessed 24 h post last heroin administration following the administration of naloxone (1 mg/kg i.p). Heroin CPP reinstatement was tested following a single dose of heroin (10 mg/kg i.p.) administration. Striatal interleukin 6 (IL-6) and tumor necrosis factor alpha (TNF-α) were quantified (ELISA) after naloxone-precipitated heroin withdrawal. Compared to the vehicle, the heroin-administered rats spent significantly more time in the heroin-paired chamber (*p* < 0.0001). Concomitant administration of resveratrol and quercetin prevented the acquisition of heroin CPP, while resveratrol, quercetin, and magnolol blocked heroin-triggered reinstatement. Magnolol, quercetin, and β-catechin blocked naloxone-precipitated heroin withdrawal and increased striatal IL-6 concentration (*p* < 0.01). Resveratrol administration was associated with significantly higher withdrawal scores compared to those of the control animals (*p* < 0.0001). The results of this study show that different polyphenols target specific behavioral domains of heroin addiction in a CPP model and modulate the increase in striatal inflammatory cytokines TNF-α and IL-6 observed during naloxone-precipitated heroin withdrawal. Further research is needed to study the clinical utility of polyphenols and to investigate the intriguing finding that resveratrol enhances, rather than attenuates naloxone-precipitated heroin withdrawal.

## 1. Introduction

Heroin is a highly addictive illicit opiate that represents one of the main contributors to the global burden of illness [1]. In the United States alone, heroin use has reached epidemic proportions, affecting about 1.6% of the population aged 12 or older [2,3,4]. In Egypt, one study from the Poison Treatment Center including all cases of acute substance intoxication between 2015–2019 reported that opiate (tramadol) was the most common substance of exposure, and the greatest cause of fatality [5].

Current pharmacological treatment options for heroin addiction target µ opioid receptors as either full agonists, such as methadone, partial agonists, such as buprenorphine—which is also a ĸ-antagonist—or full antagonists, such as naltrexone [6,7,8]. Besides their suboptimal efficacy [9], the stigma around methadone [10] added to the complex logistics for dispensing buprenorphine [11] and the problem of diversion [12]; all these factors limit the patient availability of these medications and highlight the urgent need for novel, non-opioid, pharmacological agents to treat different stages of heroin addiction.

Illicit drug use, including opiates, is associated with dysregulated immune signaling [13,14], with reports of both activation and suppression of inflammatory cytokines. Morphine administration, in one study, increased microglial release of central inflammatory mediators, such as tumor necrosis factor (TNF)-alpha and interleukin (IL)-6 [15,16], and plasma IL-6 was significantly higher in methadone-maintained heroin users compared to healthy control subjects [17]. On the other hand, heroin and other opiates suppress the microglial secretion of TNF-α [14] This immunomodulatory effect of opiates is not mediated by all opioid receptors, and other signaling pathways play a significant role in opioid addiction behaviors [14,18,19].

Peroxisome proliferator-activated receptor-alpha (PPAR-α) is known to regulate cellular inflammatory response [20,21], and polyphenols, such as resveratrol, quercetin, magnolol, and β-catechin, found in many different plants [22,23,24,25], possess immune-modulatory properties, likely through the activation of PPAR-α [26,27,28,29], and their potential efficacy for treating opioid use disorder seems to be promising.

Yunusoglu et al. examined the effect of resveratrol on alcohol-induced conditioned place preference (CPP) in mice. Pretreatment with resveratrol, dose dependently, impaired ethanol preference acquisition, reinstating and facilitating the extinction of alcohol CPP [30]. Furthermore, Singh et al., showed that repeated administration of another polyphenol, quercetin, attenuated the development of tolerance to the analgesic effect of morphine and suppressed naloxone-precipitated withdrawal [31]. Quercetin pretreatment 30 min before ethanol administration in a CPP paradigm attenuated acquisition and reinstatement and accelerated the extinction of ethanol-CPP [32]. Moreover, the effects of quercetin and β-catechin on naloxone-precipitated withdrawal were tested in vitro. Both quercetin and catechin, injected into the guinea-pig ileum 10 min before morphine, were capable of blocking naloxone-induced contracture after exposure to morphine in a concentration-dependent fashion [33]. Taken together, there is evidence that polyphenols attenuate behavioral manifestations of ethanol, morphine, and methamphetamine administration and attenuate drug-induced activation of certain inflammatory cytokines. In this study, we aimed to examine the efficacy of four different natural polyphenols in blocking behavioral manifestations of heroin administration using a conditioned place preference (CPP) paradigm.

Conditioned place preference (CPP) is a valid and reliable method used to assess the rewarding properties of various drugs of abuse [34], such as drug-paired craving and relapse [35].

## 2. Methods

### 2.1. Animals

Adult inbred male Sprague Dawley rats weighing 180 to 210 g (n = 88) were used for the experiments. The study was conducted according to the guidelines of the Declaration of Helsinki and approved by the Animal Care and Use Committee of the Egyptian National Research Center (protocol #19-220/20/11/2019). The rats were housed in standard plastic cages, with 4 animals/cage, in a controlled environment (temperature, 25–26 °C, humidity, 45–65%, and 12 h dark: light cycle, with lights on at 7:00 a.m.) and food/water were provided ad libitum.

### 2.2. Groups

Rats were randomly assigned to two cohorts. The first cohort (n = 56) was used to establish CPP to test naloxone-precipitated withdrawal, and to assay striatal IL-6 and TNF-α concentration. The second cohort (n = 32) was established to test heroin-triggered reinstatement. Rats in the first cohort were assigned to one of five groups: (1) control (n = 8), (2) heroin + quercetin (n = 12), (3) heroin + resveratrol (n = 12), (4) heroin + β-catechin (n = 12), and (5) heroin + magnolol (n = 12). Rats in the second cohort were assigned to one of six groups (n = 8 each): (1) vehicle negative control, (2) heroin positive control, (3) quercetin, (4) resveratrol, (5) β-catechin, and (6) magnolol.

### 2.3. Drugs

Heroin was provided from the Criminal Justice Laboratories under the permission of the Ministry of Justice, Cairo, Egypt. Resveratrol (Doctor’s best, CA, USA), quercetin (Naturebell, Chino, CA, USA), β-catechin (Puritan’s Pride, Holbrook, NY, USA), and magnolol (Nutricrafters, Sparks, NV, USA) were obtained from a local pharmacy as dietary supplements. We discarded the capsule, which contains inactive ingredients such as cellulose, gelatin, rice flour, silica, and maltodextrin. The content of the capsule contains the active substance only. We dissolved the active ingredient in distilled water to the required concentration to be administered orally at 1 mL doses.

### 2.4. Behavioral Study

**Figure 1:**
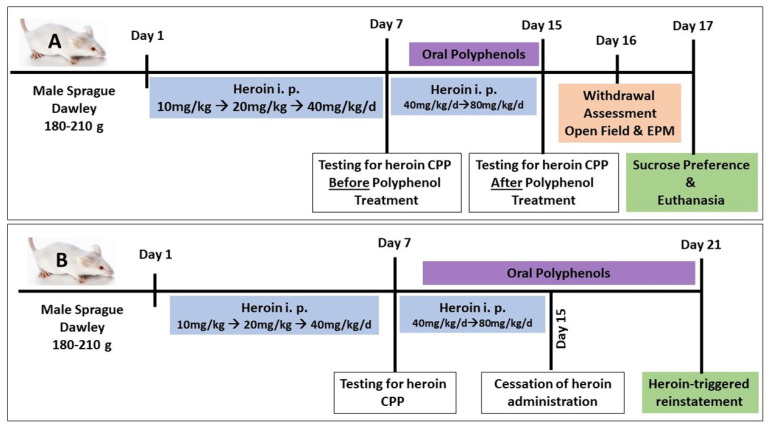
Study design: (**A**) naloxone-precipitated opiate withdrawal cohort; (**B**) heroin-triggered reinstatement cohort.

(A) Conditioned place preference (first cohort)
  - Habituation: Before the start of the procedure, the rats were habituated to the place preference laboratory room for one hour. During the preconditioning phase (1 day), the animals were allowed to freely explore the whole apparatus for 15 min. The time spent in each chamber, while the door is open, was recorded (unconditioned preference), and then the animals were returned to their home cages.
  - Establishing CPP: The following day, control rats received saline (0.5 mL/kg, i.p.), while heroin-primed rats received heroin in the least preferred chamber in escalating doses, starting from 10 mg/kg, i.p. up to 80 mg/kg daily for 14 consecutive days (10 mg/kg/d for 4 days, then 20 mg/kg/d for 4 days, then 40 mg/kg/d for 4 days, then 80 mg/kg/d for 2 days). The rats received treatments for heroin CPP starting on day 8. Animals were administered polyphenols (quercetin 50 mg/kg/d [36], or β-catechin 100 mg/kg/d [37], or resveratrol 30 mg/kg/d [38], p.o., or magnolol 50 mg/kg/d, p.o. [39]) or distilled water (1 mL) through oral gavage for 7 consecutive days starting at day 8 of heroin administration.
  - Testing for heroin CPP and testing the efficacy of polyphenols in blocking heroin CPP: On day 15, the animals were tested for heroin preference during 10 min of free access to both chambers. The percentage of time spent in the drug-paired chamber was recorded manually by a blinded observer in real time.
  - Testing for the efficacy of polyphenols in attenuating naloxone-precipitated heroin withdrawal: On day 16 after testing for heroin CPP, rats were challenged with naloxone (1 mg/kg, i.p.) after 24 h of the last heroin dose (between 8:00 a.m. and 12:00 p.m.) to precipitate withdrawal, and they were observed in a transparent cylinder arena for a 30 min test period to detect withdrawal symptoms; scores were recorded manually in real time by a blinded observer [40]. Specific withdrawal signs, including jumping, wet dog shakes, head shakes, teeth chattering, tremors, and rearing movements, were counted during every 5 min observation. Irritation, piloerection, salivation, diarrhea, and grooming were observed and scored on a four-point scale: 0 = absent; 1 = mild; 2 = moderate; 3 = severe. The scores for each time period were combined [1].
  - Testing for the effect of naloxone-precipitated withdrawal on spontaneous locomotor activity using an open field: On day 16, the rats were screened in an open field at the end of the experiment for 5 min (Fernandes et al., 2012). Each rat was placed in the center of the field (100 × 100 cm white box), and the number of squares crossed, as well as the vertical rears, were monitored by a blind observer in real time.
  - Testing for the effect of naloxone-precipitated withdrawal on anxiety using elevated plus maze (EPM): Following the open field experiment, the rats were tested for anxiety using EPM. The maze was raised 40 cm off the floor with two equal crossed arms (10 cm wide and 100 cm long), and one arm was closed by 30 cm high walls. Animals were placed at the intersection facing one open arm and allowed to freely move; the time spent in the open or closed arm, in addition to the number of entries into any of the arms, was recorded in real time by a blinded observer. Maze sessions of 5 min each were held after 90 min of naloxone-induced withdrawal [41].
  - Testing for the effect of naloxone-precipitated withdrawal on sucrose preference: On day 17 (next day of naloxone-precipitated withdrawal), the rats were deprived of food for 12 h, starting at 8:00 p.m. and continuing until 8:00 a.m. the next day, and were placed in individual cages and provided two regular 200 mL bottles: one containing 3% sucrose solution and the other containing tap water. On the next day (8:00 a.m.), the volumes of sucrose-containing water and plain water were recorded after 24 h. Sucrose intake was calculated: sucrose preference = sucrose intake/total intake (sucrose + water intake) × 100 (28).
  - Euthanasia and brain tissue collection: The rats were euthanized by decapitation under light anesthesia after the end of the sucrose preference test on day 17. Brain tissues were dissected and stored at −80 °C for molecular assay (Figure 1A).
(B) Heroin-triggered-reinstatement (second cohort)

After establishing heroin CPP as described above, the rats were kept in normal housing conditions for 6 days, without heroin exposure. On the day 7, the rats were challenged in the CPP drug-linked chamber by a single heroin dose administration (10 mg/kg, i.p.), whereas polyphenols were administered orally from day 8 to day 21, and the last dose was administered 30 min before heroin challenge. Reinstatement was assessed as the time spent in the drug-linked chamber (Figure 1B). At the end of the experiment, the rats were euthanized, and brain tissue was collected and stored for further studies.

### 2.5. Striatal TNF-α and IL-6 Assay

The micro-ELISA plate was pre-coated with an antibody specific to Rat TNF-α or IL-6 (Elabscience^®^, Houston, TX, USA). After adding samples/standards, a biotinylated detection antibody specific for Rat TNF-α or IL-6 and Avidin-Horseradish Peroxidase (HRP) conjugate were added successively to each well and incubated. The optical density of TNF-α or IL-6 conjugated with the biotinylated detection antibody was measured spectrophotometrically at a wavelength of 450 nm using a plate reader (BMG Labtech, FLUOstar Omega, Ortenberg, Germany). The OD value is proportional to the concentration.

### 2.6. Statistical Analysis

Results are expressed as the mean ± SEM. Graphpad Prism software was used to perform statistical analysis, employing one way ANOVA, followed by Dunnett’s multiple comparisons test. Statistical significance was considered at *p* < 0.05.

## 3. Results

### 3.1. Behavioral Effects

Resveratrol and quercetin attenuated the acquisition of heroin conditioned place preference

**Figure 2:**
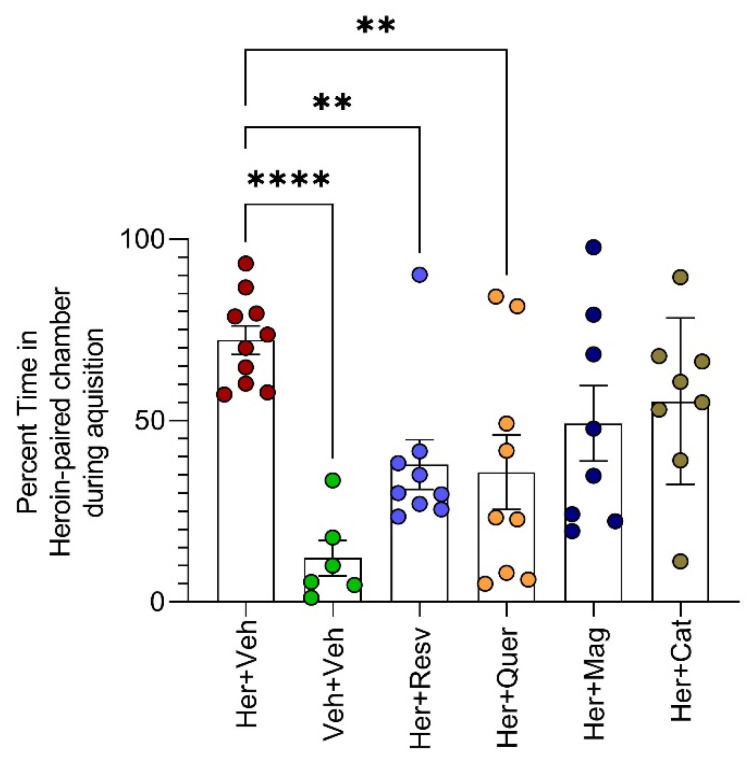
Heroin CPP acquisition is blocked by resveratrol and quercetin.

Daily heroin administration for 14 days successfully produced heroin CPP. Compared to the vehicle, heroin-administered rats spent significantly more time in the heroin-paired chamber (*p* < 0.0001) Concomitant administration of resveratrol and quercetin prevented the acquisition of heroin CPP (Figure 2).

Daily heroin administration for 14 days established heroin dependence [F(5, 44) = 6.198, *p* = 0.0002]. Heroin administered animals (compared to the vehicle) spent significantly more time in the heroin-paired chamber [mean difference = 60.06, 95% CI = 29.19 to 90.92, *p* < 0.0001]. Resveratrol and quercetin administration concomitant with heroin successfully prevented heroin preference and reduced the percentage of time spent in the heroin-paired chamber compared to the administration of heroin alone. [Her + Veh vs. Her + Resv mean difference = 34.32, 95% CI = 6.856 to 61.78, *p* = 0.009, and Her + Veh vs. Her + Quer mean difference = 36.41, 95% CI = 8.948 to 63.87, *p* = 0.005]. Neither magnolol nor β-catechin administration reduced heroin preference, according to one way ANOVA, followed by Dunnett’s multiple comparisons test, against Her + Veh control group, n = 6–10 animals per group.

Magnolol, quercetin, and β-catechin block naloxone-precipitated heroin withdrawal, prevent rapid weight loss during withdrawal, and reduce withdrawal-associated anxiety-like behavior

**Figure 3:**
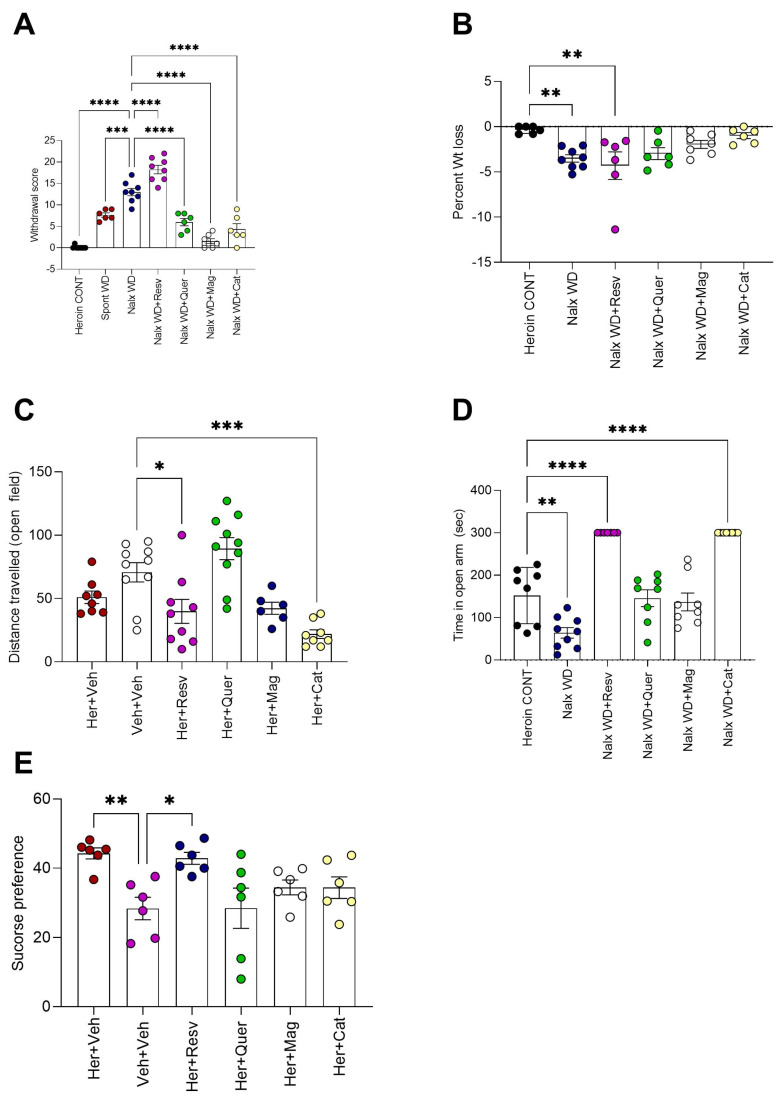
(**A**) Magnolol attenuates naloxoneprecipitated heroin withdrawal: magnolol blocked naloxone-precipitated heroin withdrawal. One-way ANOVA F(6, 42) = 70.51, *p* < 0.0001 [mean difference between heroin control and naloxone+ magnolol = −1.446, 95% CI = −4.412 to 1.519, *p* = 0.6]. Resv, Quer, and Cat all are associated with significantly higher withdrawal scores compared to those of the control animals (*p* < 0.0001 for Resv and Quer and *p* = 0.003 for Cat). Resv specifically caused more opiate withdrawal, even more than spontaneous and naloxone-precipitated withdrawals [mean difference in withdrawal scores between Resv and heroin control = −18.3, between spontaneous WD and heroin control = −7.5, and between naloxone-precipitated WD and heroin control = −12.9, according to one way ANOVA, followed by Dunnett’s multiple comparisons test against Her + Veh control group, n = 6–10 animals per group (** *p* < 0.01, *** *p* < 0.001, **** *p* < 0.0001). (**B**) Magnolol, quercetin, and β-catechin prevented the rapid wight loss associated with naloxone-precipitated heroin withdrawal: significant weight loss during naloxone-precipitated opiate withdrawal according to one-way ANOVA F(5, 33) = 4.605, *p* = 0.002 [mean difference in % body weight between heroin control and naloxone-precipitated withdrawal = 3.180, 95% CI = 0.6483 to 5.712, *p* = 0.009]. Mag [mean difference in % body weight between heroin control and naloxone-precipitated withdrawal + magnolol = 1.629, 95% CI = −0.9795 to 4.237, *p* = 0.3], Quer [mean difference in % body weight between heroin control and naloxone-precipitated withdrawal+ quercetin = 2.648, 95% CI = −0.05820 to 5.355, *p* = 0.056], and Cat [mean difference in % body weight between heroin control and naloxone-precipitated withdrawal + catechin = 0.6467, 95% CI = −2.060 to 3.353, *p* = 0.9] prevented weight loss, while Resv was associated with significant weight loss [mean difference in % body weight between heroin control and naloxone-precipitated withdrawal+ resveratrol = 3.973, 95% CI = 1.267 to 6.680, *p* = 0.002] by one way ANOVA followed by Dunnett’s multiple comparisons test against Her + Veh control group, n = 6–10 animals per group (** *p* < 0.01, *** *p* < 0.001, **** *p* < 0.0001). (**C**) Heroin administration did not cause reduction in voluntary locomotor activity as measured in an open field: One-way ANOVA F(5, 45)= 11.16, *p* < 0.0001 [Veh + Veh vs. Her + Veh mean difference = 19.70, 95% CI = −7.256 to 46.66, *p* = 0.2]. However, both Resv [Veh + Veh vs. Her + Resv mean difference = 30.81, 95% CI = 4.700 to 56.92, *p* = 0.01] and cat [Veh + Veh vs. Her + Cat mean difference = 48.83, 95% CI = 21.87 to 75.78, *p* = 0.0001] caused significant reduction in distance traveled by one way ANOVA followed by Dunnett’s multiple comparisons test against Her + Veh control group, n = 6–10 animals per group (* *p* < 0.1, ** *p* < 0.01, *** *p* < 0.001, **** *p* < 0.0001). (**D**) Magnolol and quercetin reduce anxiety-like behavior during naloxone-precipitated withdrawal as measured by time spent in open arm of elevated plus maze: Naloxone-precipitated withdrawal is associated with significant reduction in time spent in open arm compared to heroin controls by one-way ANOVA F(5, 42) = 34.75, *p* < 0.0001, [mean difference in time between heroin control and naloxone-precipitated withdrawal = 87.86, 95% CI = 29.41 to 146.3, *p* = 0.001]. Mag [mean difference in time between heroin control and naloxone-precipitated withdrawal + Mag = 14.88, 95% CI = −45.27 to 75.02, *p* = 0.9] and Quer [mean difference in time between heroin control and naloxone-precipitated withdrawal + Quer −6.125, 95% CI = −54.02 to 66.27, *p* = 0.9] increased the time in open arm to be non-significantly different from heroin control animals. However, Resv [mean difference in time between heroin control and naloxone-precipitated withdrawal + Resv = −148.3, 95% CI = −210.5 to −85.99, *p* < 0.0001] and Cat [mean difference in time between heroin control and naloxone-precipitated withdrawal + Cat = −148.3, 95% CI = −208.4 to −88.10, *p* < 0.0001] were associated with a significant increase in open arm time compared to controls by one way ANOVA followed by Dunnett’s multiple comparisons test against Her + Veh control group, n = 6–8 animals per group. (** *p* < 0.01, *** *p* < 0.001, **** *p* < 0.0001). (**E**) Quercetin, magnolol, and β-catechin attenuate heroin-induced sucrose preference: Heroin administration was associated with significant increase in sucrose preference test by one-way ANOVA [F(5, 30) = 4.300, *p* = 0.004, heroin vs. vehicle mean difference = 15.84, 95% CI = 28.18 to 3.495, *p* = 0.008]. Resv did not reduce the increase in heroin-induced sucrose preference Resv vs. Vehicle mean difference = 14.44, 95% CI = 26.78 to 2.092, *p* = 0.017]. Quer, Mag and Cat, on the other hand attenuated heroin-induced increase in sucrose preference by one way ANOVA followed by Dunnett’s multiple comparisons test against Her + Veh control group, n = 8–10 animals per group (* *p* < 0.1, ** *p* < 0.01, *** *p* < 0.001, **** *p* < 0.0001).

Opiate withdrawal manifestations measured 24 h post last heroin administration showed significantly higher scores in both spontaneous and naloxone-induced withdrawal (*p* < 0.0001 for both, Figure 3A) associated with significant weight loss (≈3% of body weight in 24 h, Figure 3B). Magnolol blocked naloxone-precipitated heroin withdrawal (*p* < 0.0001 Figure 3A), prevented weight loss (1.6% of body weight compared to heroin control animals, *p* = 0.3 vs. Her + Veh, Figure 3B), and increased the time spent in the EPM open arm in a non-significantly different manner from the heroin control animals (*p* = 0.9, Figure 3D) (** *p* < 0.01, *** *p* < 0.001, **** *p* < 0.0001).

Quercetin and β-catechin are associated with significantly lower withdrawal scores compared to naloxone-precipitated withdrawal [mean difference in withdrawal scores between naloxone vs naloxone + quercetin = 7.00, 95% CI = 3.906 to 10.09 *p* < 0.0001 and naloxone vs naloxone + β-catechin = 8.667, 95% CI = 5.572 to 11.76 *p* < 0.0001, Figure 3A]. In addition, both quercetin and β-catechin prevented naloxone precipitated withdrawal-induced weight loss [mean difference in % body weight between heroin control and naloxone+ quercetin = 2.648, 95% CI = −0.05820 to 5.355, *p* > 0.05, and between heroin control and naloxone + B-catechin = 0.6467, 95% CI = −2.060 to 3.353, *p* > 0.05, Figure 3B]. β-catechin treatment was associated with a significant reduction in distance traveled in the open field (*p* < 0.0001, Figure 3C). Quercetin, magnolol, and β-catechin all attenuated sucrose preference associated with opiate withdrawal (*p* > 0.05, Figure 3E).

### 3.2. Resveratrol Worsens Naloxone-Induced Heroin Withdrawal

On the other hand, resveratrol showed a higher withdrawal score compared to naloxone [mean difference between naloxone vs naloxone + resveratrol = −5.2, 95% CI = −8.115 to −2.385 *p* < 0.0001, Figure 3A], and was associated with significant weight loss [mean difference in % body weight between heroin control and naloxone +resveratrol = 3.973, 95% CI = 1.267 to 6.680, *p* < 0.01, Figure 3B]. Resveratrol inhibited the distance traveled in the open field (*p* < 0.01 Figure 3C) but did not affect sucrose preference associated with heroin (*p* < 0.05, Figure 3E).

Resveratrol, quercetin, and magnolol prevent heroin-triggered reinstatement.

**Figure 4:**
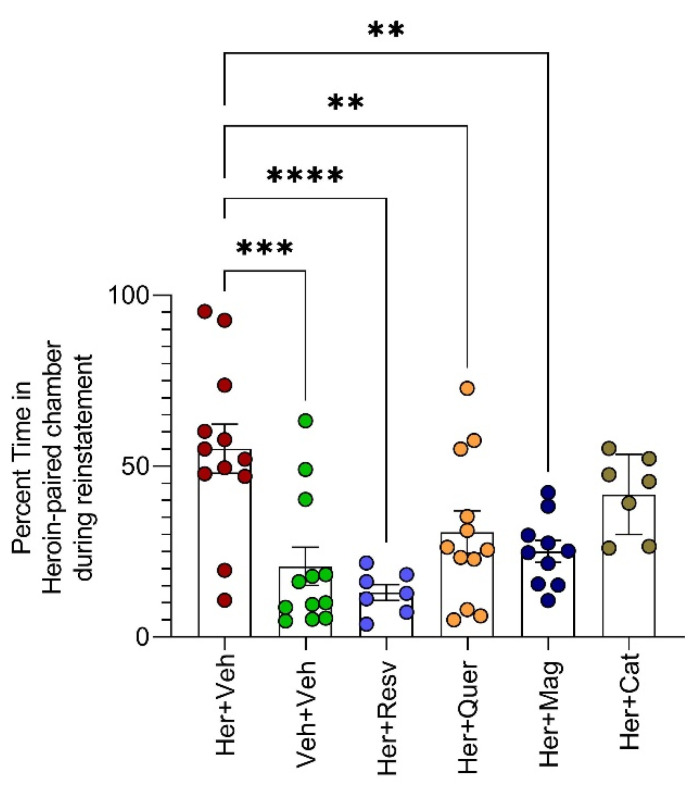
Resveratrol, quercetin, and magnolol prevent heroin-triggered reinstatement.

A single heroin dose (10 mg/kg) triggered reinstatement, as evidenced by the significant increase in the percentage of time spent in the heroin-paired chamber compared to the vehicle (*p* = 0.0001). Animals subjected to heroin administration along with resveratrol, quercetin, and magnolol spent significantly less time in the heroin-paired chamber compared to the heroin+ vehicle group (*p* < 0.01 each, Figure 4).

A single heroin dose triggered reinstatement, as evidenced by the percentage of time spent in the heroin-paired chamber, as assessed by one-way ANOVA F(5, 54) = 6.983, *p* < 0.0001, [mean difference in percentage of time spent in heroin-paired chamber between heroin and vehicle groups = 34.40, 95% CI = 14.99 to 53.82, *p* = 0.0001]. Resveratrol [mean difference in percentage of time in heroin-paired chamber between heroin and heroin + resveratrol groups = 42.06, 95% CI = 19.45 to 64.68, *p* < 0.0001], quercetin [mean difference in percentage of time in heroin-paired chamber between heroin and heroin + quercetin groups = 24.36, 95% CI = 4.947 to 43.78, *p* = 0.008], and magnolol [mean difference in percentage of time in heroin-paired chamber between heroin and heroin + magnolol groups = 30.04, 95% CI = 9.682 to 50.41, *p* = 0.001] all prevented heroin-triggered reinstatement. However, β-catechin did not [mean difference in percentage of time in heroin-paired chamber between heroin and heroin + β-catechin groups = 13.40, 95% CI = −9.222 to 36.01, *p* = 0.4], according to one way ANOVA followed by Dunnett’s multiple comparisons test against Her + Veh control group, n = 7–11 animals per group (** *p* < 0.01, *** *p* < 0.001, **** *p* < 0.0001).

### 3.3. Molecular Effects

**Figure 5:**
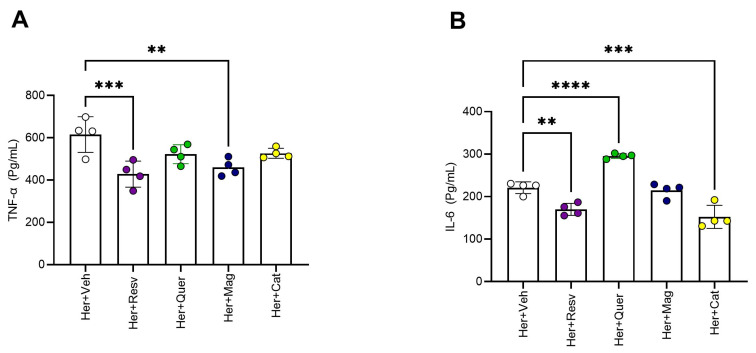
The effect of polyphenols on striatal TNF α and IL-6 and concentration. (**A**) Resveratrol and magnolol attenuate heroin-induced increase in striatal TNF-α concentration: Significant differences in striatal TNF-α concentrations were evident by one-way ANOVA [F(4, 15) = 6.885, *p* < 0.01]. Dunnett’s multiple comparisons test against Her + Veh control group (n = 4) showed significant effect of resveratrol [mean difference in striatal TNF-α concentration between heroin and heroin+ resveratrol groups = 187.4, 95% CI = 81.40 to 293.4, *p* < 0.001], and magnolol [mean difference in striatal TNF-α concentration between heroin and heroin+ magnolol groups = 155.9, 95% CI = 49.84 to 261.9, *p* < 0.01] on TNF-α concentration. (**B**) Resveratrol and β-catechin attenuate, while quercetin accentuates, heroin-induced increase in striatal IL-6 concentration: Significant differences in striatal IL-6 concentrations were evident by one-way ANOVA [F(4, 15) = 42.74, *p* < 0.0001]. Dunnett’s multiple comparisons test against Her + Veh control group (n = 4) showed significant effect of resveratrol [mean difference in striatal IL-6 concentration between heroin and heroin+ resveratrol groups = 51.11, 95% CI = 18.24 to 83.98, *p* < 0.01], quercetin [mean difference in striatal IL-6 concentration between heroin and heroin + quercetin groups = −74.66, 95% CI = −107.5 to −41.78, *p* < 0.0001], and β-catechin [mean difference in striatal IL-6 concentration between heroin and heroin+ β-catoctin groups = 68.69, 95% CI = 35.82 to 101.6, *p* < 0.001] on IL-6 concentration (** *p* < 0.01, *** *p* < 0.001, **** *p* < 0.0001).

Resveratrol and magnolol attenuated the heroin-induced increase in striatal TNF-α concentration (*p* < 0.001, *p* < 0.01 respectively), while resveratrol and β-catechin attenuated the heroin-induced increase in striatal IL-6 concentration (*p* < 0.01, *p* < 0.001 respectively). On the other hand, quercetin accentuated IL-6 concentration (*p* < 0.0001) (Figure 5).

## 4. Discussion

The results of this study show that different polyphenols target specific behavioral domains of heroin addiction in a CPP model and modulate the increase in striatal inflammatory cytokines TNF-α and IL-6 observed during naloxone-precipitated heroin withdrawal.

### 4.1. Quercetin Abolished Heroin Dependence Acquisition and Inhibited Reinstatement Attributed to Anti-Inflammatory Effects

Specifically, quercetin blocked the acquisition of heroin CPP, reduced withdrawal manifestations and heroin-triggered reinstatement, prevented heroin-induced sucrose preference, and accentuated the heroin-induced increase in striatal IL-6 concentration. Interestingly, quercetin prevented certain aspects of naloxone-precipitated heroin withdrawal, such as rapid wight loss and anxiety-like behavior.

Our results are in accordance with those of Singh et al., who reported that repeated administration of quercetin (25 and 50 mg/kg) for 9 days suppressed naloxone-precipitated morphine (10 mg/kg) withdrawal [31]. On the other hand, quercetin inhibited nicotine-triggered CPP reinstatement [42], alleviated METH-induced anxiety-like behavior in mice, attenuated the activation of astrocytes, and reduced the levels of IL-1beta and TNF-α, but not IL-6 [43]. In addition, quercetin (10, 30 and 100 mg/kg i.p.) pretreatment 30 min before ethanol administration in a CPP paradigm attenuated the acquisition and reinstatement and accelerated the extinction of ethanol-CPP [32]. Additionally, quercetin reversed morphine tolerance, attenuated morphine withdrawal expression in mice [44], and prevented ethanol-induced withdrawal somatic manifestations [36]. Taken together, it seems that quercetin is effective in blocking the acquisition and preventing the reinstatement of certain substances. Further studies are needed to investigate these specific aspects of quercetin before proposing proof of concept pilot studies in human heroin users.

### 4.2. Resveratrol Blocked Heroin Acquisition and Drug-Induced Reinstatement Effectively, but Accentuated Withdrawal Manifestations

The current results show that resveratrol, like quercetin, blocked the acquisition of heroin CPP and heroin-triggered reinstatement. However, it was associated with significantly higher withdrawal scores compared to heroin control, but unlike quercetin, resveratrol attenuated the heroin-induced increase in striatal TNF-α and IL-6 concentrations. Our results are the first, to the best of our knowledge, to report the effect of resveratrol on heroin addiction. Few studies have examined the effects of resveratrol on the attenuating behavioral manifestations of other substance. Yunusoglu et al. examined the effect of resveratrol on alcohol-induced conditioned place preference (CPP) in mice. Pretreatment with resveratrol (25, 50, and 75 mg/kg, i.p.) 30 min prior to ethanol administration impaired acquisition, and reinstatement of alcohol induced CPP and facilitated the extinction of alcohol CPP [30]. Moreover, pretreatment with resveratrol (10 or 100 mg/kg i.p.) remarkably attenuated methamphetamine (METH)-induced memory impairment in mice and reversed METH-induced oxidative damage and apoptosis [45]. As such, the current literature, including our results, suggest that resveratrol is also effective in blocking acquisition and preventing reinstalment of certain substances, but again, we observed a worsening of naloxone-precipitated heroin withdrawal. Calleri et al. [46] showed the antagonistic activity of resveratrol on PPARα and PPARγ. Further studies are needed to investigate whether the efficacy of resveratrol in blocking the acquisition of heroin CPP, heroin-triggered reinstatement, or its side effects in accentuating naloxone-induced heroin withdrawal is mediated through PPARα or PPARγ.

### 4.3. β-Catechin Blocked Reinstatement, but Not Acquisition of Heroin Dependence and Reduced the Withdrawal Manifestations

In the case of β-catechin, our results show that it failed to block the acquisition of heroin CPP or to attenuate naloxone-precipitated heroin withdrawal, but it prevented heroin-triggered reinstatement. Shutto et al. reported that resveratrol (40 mg/kg s.c.) enhanced the acute effect of cocaine on locomotor activity [47]. The authors speculated that this effect could be due to resveratrol enhancing dopamine neurotransmission through the inhibition of MAO-A and MAO-B. Further studies are needed to investigate these underlying mechanisms and to determine if indeed certain polyphenols inhibit MAO-A and MAO-B or activate GABA_(A)_ receptors (as reported for quercetin), then the efficacy of these compounds in the treatment of depression and anxiety should be examined.

### 4.4. Magnolol Blocked Heroin-Induced Reinstatement, but Did Not Affect Acquisition and Withdrawal

Unlike quercetin and resveratrol, magnolol failed to block the acquisition of heroin CPP, but it successfully prevented heroin-triggered reinstatement. In addition, magnolol attenuated naloxone-precipitated heroin withdrawal, prevented rapid wight loss and anxiety-like behavior associated with naloxone-precipitated heroin withdrawal, and attenuate heroin-induced increase in striatal TNF-α concentration. More studies focused on this unique property of magnolol in attenuating heroin withdrawal manifestations are required.

### 4.5. Could Polyphenols Act through Dopaminergic Mechanisms?

Shutto et al. reported that resveratrol (40 mg/kg s.c.), enhanced the acute effect of cocaine on locomotor activity [47]. The authors speculated that this effect could be due to resveratrol enhancing dopamine neurotransmission through the inhibition of MAO-A and MAO-B. Further studies are needed to investigate these underlying mechanisms and if indeed certain polyphenols inhibit MAO-A and MAO-B or activate GABA_(A)_ receptors (as reported for quercetin), then the efficacy of these compounds in the treatment of depression and anxiety should be examined.

PPAR-γ agonists can block rewarding properties of drugs through stimulating the mesolimbic dopaminergic neurotransmission [48,49]. The examined polyphenols are known to modulate PPAR-γ; thus, the acquisition blocking action of quercetin and resveratrol may be linked to dopamine transmission modulation. Quercetin was reported to increase the dopaminergic neuron density in the striatum of experimental PD in mice [50,51], and resveratrol exerted anti-depressant effects through modulating dopamine and serotonin, as shown in a previous report [52].

### 4.6. Could the Current Results Be Related to an Anti-Inflammatory Mechanism?

At the molecular level, we examined two neuroinflammatory markers in the striatum, IL-6 and TNF-α, following naloxone-induced heroin withdrawal. Our results show that magnolol, which successfully blocked withdrawal manifestations, attenuated the heroin-induced increase in striatal TNF-α, while quercetin was also associated with an increase in striatal IL-6 concentration. Magnolol attenuates the increase in pro-inflammatory cytokines such as IL-1β, IL-6 and TNF-α [53,54,55,56,57,58,59]. In addition, magnolol reduces glutamate-induced cytotoxicity in neuronal cell cultures [60], restores blood–brain barrier integrity, and reduces ischemia-associated brain edema [54], suggesting a neuroprotective property for magnolol against post ischemic stroke [61]. Several studies have shown its efficacy in reversing depressive-like behaviors in animal models using the sucrose preference test, the forced swim test [53,62], olfactory bulbectomy [63], and chronic unpredictable mild stress [64].

On the other hand, resveratrol attenuated striatal TNF-α, and β-catechin attenuated striatal IL-6 concentrations. Both resveratrol and β-catechin were of limited value in attenuating withdrawal manifestations. These results highlight the complexity of the neuroimmunological changes that take place during heroin use and the effects of different polyphenols on immune markers.

### 4.7. The Role of Immunomodulatory Mechanisms

Heroin and other exogenous opiates exert neuromodulatory effects through both immune suppression and activation, depending on the stage of drug use [65]. Acute morphine administration and morphine withdrawal both cause immune suppression [66]. Significant reduction in the response of T-lymphocytes to phytohemagglutinin challenge during acute withdrawal in heroin addicts has been reported [67]. Chronic heroin self-administration in rats produced a significant increase in lipopolysaccharide (LPS)-induced tumor necrosis factor-alpha (TNF-α) [68]. A similar increase in TNF-α, along with a marked elevation in total and activated B cells and IL-8 was reported in human heroin users with HIV and hepatitis C (n = 19) compared to controls (n = 19) [69]. This immune activation, with the rapid rise in cytokines, modulates the mesolimbic dopaminergic reward network, facilitating drug dependence and also contributing to the development of the acute withdrawal state [70,71].

The effects of individual polyphenols on the immune system are also complex. For example, resveratrol modulates the immune response, with both anti-inflammatory [72,73] and immune-enhancing effects [74,75], possibly in a dose-dependent manner [76]. One study reported that resveratrol interfered with the synthesis and gene expression of proinflammatory cytokines [77] through the suppression of the nuclear factor (NF)-kappaB signaling pathway. NF-kappaB plays a significant role in augmenting the inflammatory response through the release of free radicals [78]. In addition, resveratrol inhibited the production of TNF-α and IL-12 by peritoneal macrophages and blocked the activation of the transcription factor NF-kappaB, without affecting basal NF-kappaB activity [79]. On the other hand, resveratrol enhanced the immunity recovery of immunosuppressive mice through activating the NF-kappaB pathway and upregulating the expression of serum IL-2 and TNF-α in a dose-dependent manner [38]. In healthy volunteers (n = 10), resveratrol showed a significant increase in TNF-α levels 24 h after treatment compared to the baseline [74]. As such, resveratrol seems to exert different effects based on the underlying immune status.

Quercetin also exhibits immunomodulatory activities through inhibiting the secretion of inflammatory cytokines and improving immune function [reviewed in [80,81,82,83,84]]. Quercetin significantly inhibited the production of IL-6, and TNF-α in poly IC-induced RAW 264.7 mouse macrophages [85], reduced TNF-α and IL-8 mRNA expressions in a dose-dependent manner in zebrafish [86], and improved immune function via the NF-kappaB signaling pathway triggered by TNF-α in one-day-old healthy Arbor Acre broilers. [87].

β-Catechin is a natural immune enhancer present in several plants such as green tea leaves, black grapes, and cherries [88,89,90]. One study reported that catechin inhibited the gene expression of pro-inflammatory cytokines IL-1β and IL-6, and enhanced the gene expression of anti-inflammatory cytokines IL-4 and IL-10 [91]. At a behavioral level, catechin (25, 50, and 100 mg/kg administered orally for 11 to 25 days) was associated with significant improvement in behavioral manifestations of sociability, stereotypy, anxiety, depression, novelty, repetitive, and perseverative behaviors in rodents [92]. Another study showed that β-catechin increased life-span in a senescence accelerated mouse model of aging [93].

The role of PPAR-γ activation cannot be neglected. PPAR-γ expression functions as a vital regulator in NF-κappaB-mediated inflammation [94]. It was postulated that PPAR-γ activation by agonists such as leriglitazone reduce oxidative stress and boost biogenesis and mitochondrial functionality associated with the NF-kappaB inflammatory mechanisms [95], resulting in anti-inflammatory and anti-oxidative stress regulation [96].

Clinical data relative to the use of these compounds in drug use and dependence is not present. However, quercetin, resveratrol, and catechin were investigated clinically in many other disorders such as diabetes, cancer, arthritis, or neurodegenerative diseases, while magnolol was investigated in dental and periodontal studies (clinicaltrials.gov; review [97,98,99,100,101].

## 5. Limitations

The results of this study should be viewed in light of its limitations. First, we examined only adult male rats and could not comment on the efficacy of tested compounds in adolescents or female rats. Second, we did not examine different doses or the optimal therapeutic window for the efficacy of polyphenols for different stages of drug use. Third, we investigated only IL-6 and TNF-α following naloxone-precipitated withdrawal and did not examine other neuroimmune markers during acquisition or reinstatement. Future studies are needed to expand on the current findings and examine other neuroinflammatory markers at each stage of heroin addiction. Despite these limitations, the results of this study lend more evidence to the potential therapeutic benefits of PPAR agonists in reversing certain behavioral manifestations of heroin use and highlight the immunomodulatory function of these compounds, with some concerns for worsening behavioral manifestations of heroin withdrawal. Further preclinical research is needed to gain more insight into the utility of natural polyphenols in treating heroin use disorder in human subjects.

## 6. Conclusions

Despite these limitations, our current results show that four different polyphenols, with known modulatory effects at the PPAR-γ, are effective in attenuating different aspects of heroin addiction. Quercetin and resveratrol could be effective in blocking heroin relapse, while quercetin and magnolol may be utilized in reducing the severity of heroin withdrawal. Resveratrol use during early heroin abstinence might aggravate withdrawal manifestations. Β-catechin was of limited value in opioid withdrawal, but it blocked reinstatement and relapse to heroin. Proof of concept pilot clinical trials are needed to test the potential efficacy of these compounds in treating patients with heroin use disorder.

## Data Availability

Data is contained within the article.
